# Convergent roles of BcGUN4.1, BcSG1, BcCHLH, and BcTPR4 in regulating the leaf greenness of non-heading Chinese cabbage

**DOI:** 10.1186/s43897-025-00216-5

**Published:** 2026-05-08

**Authors:** Aimei Bai, Li Gong, Xinya Wang, Huanhuan Xu, Zhenzhi Zhu, Changwei Zhang, Tongkun Liu, Xilin Hou, Ying Li

**Affiliations:** State Key Laboratory of Crop Genetics and Germplasm Enhancement and Utilization, Engineering Research Center of Germplasm Enhancement and Utilization of Horticultural Crops, Ministry of Education of the P. R. China, College of Horticulture, Nanjing Agricultural University, Nanjing, 210095 Jiangsu Province China

**Keywords:** *BcGUN4.1*, *BcSG1*, *BcTPR4*, Leaf greenness, Protein–protein interaction, Non-heading Chinese cabbage

## Abstract

Non-heading Chinese cabbage (NHCC) extensively cultivated as a leafy vegetable in China, displays diverse leaf coloration, with green being an essential trait. Leaf greenness, determined by chlorophyll and carotenoid contents, is a crucial visual characteristic, and understanding its genetic and molecular mechanisms is essential for breeding. This study identified two highly homologous *BcGUN4* genes (*BcGUN4.1* and *BcGUN4.2*) in NHCC. BcGUN4.1 exhibited localization to chloroplasts and cell membranes, positively regulating pigment accumulation. Similarly, BcSG1 and BcCHLH were detected in chloroplasts and cell membranes, co-localizing with BcGUN4.1. Notably, BcGUN4.1 interacted with BcSG1 and BcCHLH in chloroplasts, while *BcSG1* functioned as a positive regulator of pigmentation. Moreover, BcGUN4.1, BcSG1, and BcCHLH interacted with BcTPR4, and BcSG1 directly bound to BcCHLH, forming a tetrameric complex in chloroplasts. *BcTPR4* contributed to pigment accumulation. Additionally, BcSG1 and BcTPR4 enhanced BcGUN4.1–BcCHLH complex activity. These findings established that BcGUN4.1, BcSG1, BcCHLH, and BcTPR4 cooperatively regulate leaf greenness in NHCC, offering novel insights into the molecular mechanisms underlying this trait.

**Supplementary Information:**

The online version contains supplementary material available at 10.1186/s43897-025-00216-5.

## Core

*BcGUN4.1*, *BcSG1*, and *BcTPR4* substantially enhanced the pigment content associated with leaf greenness in the non-heading Chinese cabbage. BcGUN4.1 demonstrated co-localization and interaction with BcSG1 and BcCHLH. In addition, BcGUN4.1, BcSG1, and BcCHLH exhibited interaction with BcTPR4, while BcSG1 also directly bound to BcCHLH. These four proteins formed a tetrameric complex, wherein BcSG1 and BcTPR4 functioned as subunits that enhanced the activity of the BcGUN4.1–BcCHLH complex.

## Gene & accession numbers

The sequence data are available on the National Center for Biotechnology Information (NCBI) website (https://www.ncbi.nlm.nih.gov/).

## Introduction

The green coloration observed in plant organs (leaves, stems, and immature fruits) is primarily controlled by the synergistic action of chlorophyll and carotenoid pigments (Amagai et al. 2022). Chlorophyll biosynthesis, which determines green pigmentation intensity, encompasses five critical stages: (1) 5-aminolevulinic acid (ALA) biosynthesis, (2) protoporphyrin synthesis, (3) chlorophyllide a formation, (4) chlorophyll a production, and (5) the chlorophyll cycle (Wang & Grimm 2021). ALA functions as the foundational precursor for tetrapyrrole biosynthesis (Ilag et al. 1994), with pathway regulation controlled by key genes including *HEMA* (Yang et al. 2023), *GSA* (Sinha et al. 2022), *Mg-chelatase H subunit* (*CHLH*), and *GENOMES UNCOUPLED 4* (*GUN4*) (Adhikari et al. 2011). Recent studies have identified additional critical regulators such as *GOLDEN2-LIKE* transcription factors (Zhang et al. 2022) and *FC2* (Fan et al. 2023), where loss-of-function mutations result in chlorophyll-deficient phenotypes. Chloroplast development represents a crucial factor in chlorophyll accumulation, as these organelles contain chlorophyll biosynthesis machinery (Pogson & Albrecht 2011). In cucumber, early developmental chlorophyll content demonstrates strong correlation with chloroplast abundance (Liu et al. 2016). Disrupted chloroplast development, often manifesting as delayed greening, has been linked to mutations in plastid-encoded RNA polymerase subunits (rpoA, rpoC1) and nuclear-encoded regulators such as SLOW GREEN 1 (SG1) (Wu & Zhang 2010; Hu et al. 2014; Huo et al. 2024a, b).

GUN4, a nuclear-encoded plastid protein, plays a dual role in chloroplast-to-nucleus retrograde signaling and chlorophyll biosynthesis (Susek et al. 1993). The protein functions as a key regulator of chlorophyll production through its interaction with Mg-CHLH, activating Mg chelatase (MgCh) activity to catalyze the conversion of protoporphyrin IX (Proto) to Mg-protoporphyrin IX (MgProto) (Larkin et al. 2003). As the catalytic core of MgCh, CHLH maintains an evolutionarily conserved functional relationship with GUN4 across cyanobacteria and higher plants (Davison et al. 2005). This critical interaction is demonstrated by chlorophyll-deficient phenotypes observed in *chlh* mutants of *Arabidopsis*, *Brassica napus*, and tomato (Adhikari et al. 2011; Zhao et al. 2020; Xu et al. 2024). Structural analyses reveal GUN4’s distinctive capacity to bind both Proto and Mg-Proto, indicating its dual function in substrate stabilization and enzymatic activation of MgCh (Larkin et al. 2003). Phenotypic analysis of Arabidopsis *gun4* mutants revealed pale-green leaves with simultaneous reductions in both Mg- and Fe-porphyrin branch activities within the tetrapyrrole biosynthetic pathway (Wilde et al. 2004; Adhikari et al. 2011). Recent research has expanded our understanding of GUN4’s regulatory network, demonstrating its interaction with chloroplast-localized MORF2 and MORF9 proteins that collectively enhance chlorophyll accumulation through MgCh modulation (Yuan et al. 2022). In addition, GUN4 forms functional complexes with key chlorophyll homeostasis regulators, including BALANCE OF CHLOROPHYLL METABOLISM 1 (BCM1) and the chloroplast signal recognition particle component cpSRP43, highlighting its central role in coordinating chlorophyll biosynthesis pathways (Wang et al. 2020; Ji et al. 2021).

Tetratricopeptide repeat (TPR)-containing proteins function as essential mediators of protein–protein interactions, facilitating the assembly of multiprotein complexes and chaperone activities crucial for chloroplast biogenesis and chlorophyll metabolism. Several TPR family members have been characterized in these processes, including *LAP1*, *REP27*, and *PYG7*, which regulate chlorophyll accumulation and photosystem II (PS II) assembly through coordinated control of chloroplast development (Peng et al. 2006; Stockel et al. 2006; Dewez et al. 2009). Additional TPR proteins, such as pTAC2 and TCP34, influence chloroplast development through specific mechanisms involving plastid transcript stabilization and RNA processing (Pfalz et al. 2006; Weber et al. 2006). SG1, a nuclear-encoded TPR protein, plays a crucial role in chloroplast differentiation. Arabidopsis *sg1* mutants exhibit severe chlorosis and impaired chloroplast maturation, demonstrating *SG1*’s essential function in early plastid development (Hu et al. 2014). Genetic analysis results showed that the *sg1 gun4* double mutant displays partial phenotypic rescue of the *sg1* delayed-greening phenotype, accumulating higher chlorophyll levels than either single mutant (Hu et al. 2014). This epistatic interaction suggests a synergistic relationship between *SG1* and *GUN4* in regulating both chlorophyll biosynthesis and chloroplast maturation.

Non-heading Chinese cabbage (*Brassica rapa* ssp. *chinensis*, NHCC), a widely cultivated leafy vegetable in China, exhibits substantial variation in leaf greenness. Its leaf color serves as both commercial quality trait and phenotypic marker for biomass accumulation, stress tolerance, and nutritional quality (Dai et al. 2018; Su et al. 2023). Genetic studies in *B. rapa* have identified multiple pigmentation-related genes, including *BrChlH*, *BrPDS*, and *BrLCYE* associated with yellow-leaf mutants (Fu et al. 2019; Huo et al. 2024a, b), and *BrFLU* mediating cold-induced leaf yellowing in specific pak-choi cultivars (Wang et al. 2021). Previous genetic mapping revealed two major quantitative trait loci (*GlcA07.1* and *GlcA09.1*) governing leaf greenness in NHCC. Functional characterization identified *BcGUN4.1* as the candidate gene for *GlcA07.1*, while *GlcA09.1* was associated with *BcSG1* (Bai et al. 2024). Molecular markers developed from *BcGUN4.1* promoter variations and *BcSG1* coding sequence (CDS) polymorphisms effectively distinguished leaf greenness across 73 cultivars (Bai et al. 2024). Complementary evidence from yellow-core ‘Wucai’ leaves demonstrating reduced *GUN4* expression, along with established genetic interactions between *GUN4* and *SG1* (Hu et al. 2014; Xie et al. 2019), further supports their involvement in chlorophyll regulation. However, the precise molecular mechanisms of *BcGUN4.1* and *BcSG1* remain to be fully elucidated. Comprehensive functional characterization through targeted mutagenesis or transgenic approaches, combined with systematic analysis of their regulatory networks, is essential to decipher the genetic basis of leaf greenness in NHCC.

This study systematically investigated the functional roles of *BcGUN4.1* and *BcSG1*, and their regulatory interactions in controlling leaf greenness in NHCC. Heterologous complementation in Arabidopsis, transient overexpression, and virus-induced gene silencing (VIGS) were used to validate the functions of *BcGUN4.1* and *BcSG1* in NHCC. In addition, the regulatory network between BcGUN4.1 and BcSG1 was computationally predicted and experimentally verified using yeast two-hybrid (Y2H), bimolecular fluorescence complementation (BiFC), firefly luciferase (LUC) complementation imaging (LCI), and pull-down assays. The findings provide insights into the molecular mechanisms regulating chlorophyll biosynthesis and chloroplast development in NHCC. The identified genetic components and their interaction networks represent valuable targets for precision breeding programs aimed at enhancing leaf quality traits in *Brassica* crops.

## Results

### *BcGUN4.1* positively regulates chlorophyll and carotenoid accumulation

**Fig. 1:**
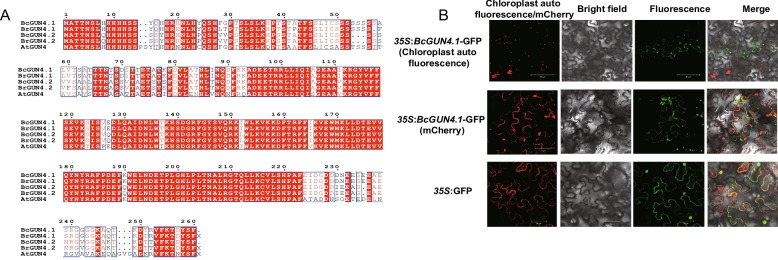
Identification and subcellular localization analysis of BcGUN4.1. **A** Amino acid sequence alignment analysis of BcGUN4. **B** Subcellular localization analysis of BcGUN4.1

Genome-wide analysis identified two GUN4 paralogs (BcGUN4.1 and BcGUN4.2) in NHCC (Fig. 1A). Comparative sequence analysis revealed high evolutionary conservation of BcGUN4 proteins, with BcGUN4.1 and BcGUN4.2 showing 87% amino acid sequence identity (Fig. 1A). Building on previous identification of *BcGUN4.1* as a key regulator of leaf pigmentation (Bai et al. 2024), subcellular localization assays demonstrated dual targeting of BcGUN4.1 to both chloroplasts and cell membranes (Fig. 1B).

**Fig. 2:**
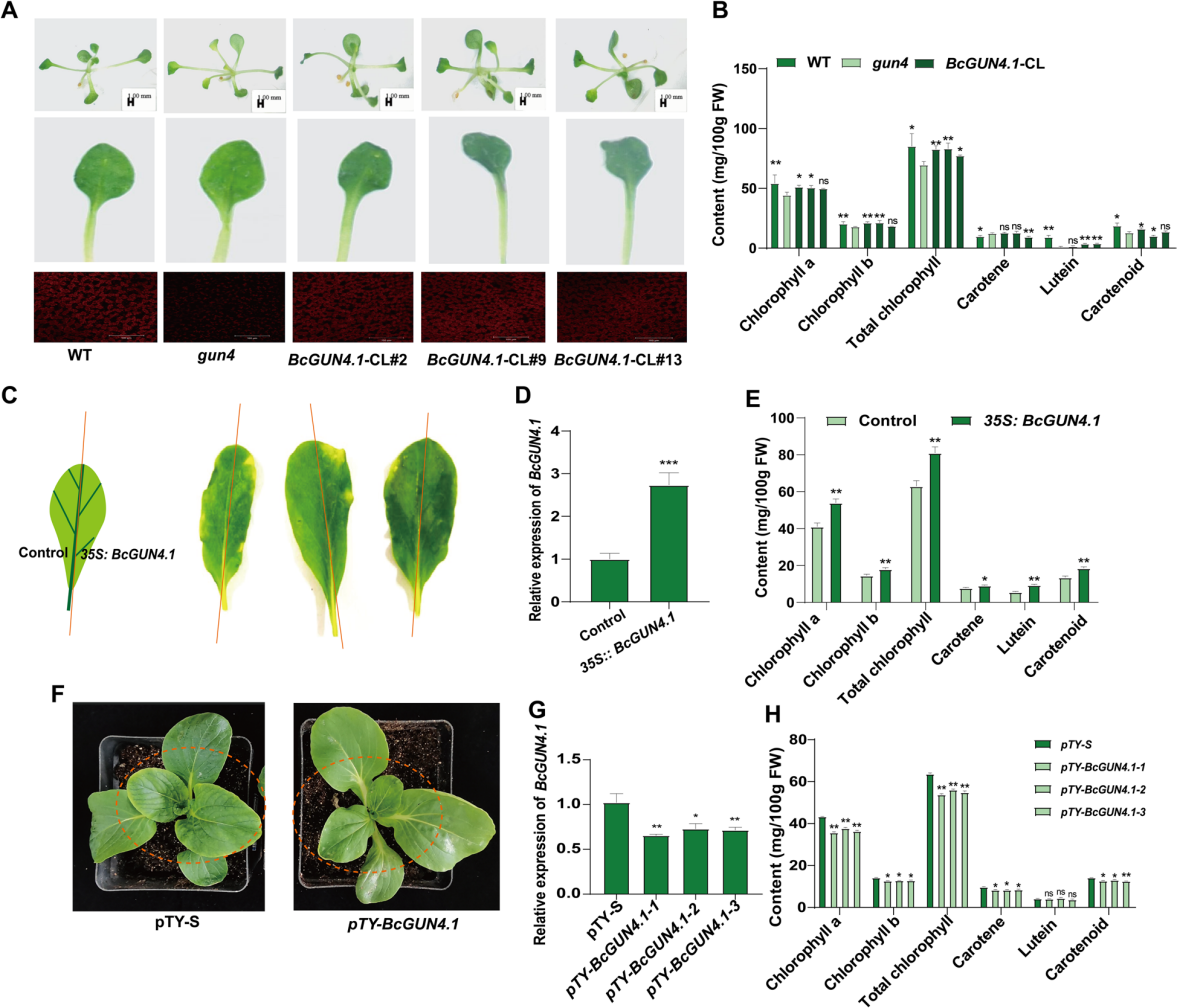
Functional characterization of *BcGUN4.1* demonstrates its role in regulating leaf greenness. **A** Phenotypic comparison of WT, *gun4* mutant, and *BcGUN4.1*-CL *Arabidopsis* plants. **B** Quantification of chlorophyll and carotenoid contents of WT, *gun4* mutant, and complementation lines at identical developmental stages. **C** Transient overexpression of *35S:BcGUN4.1* in NHCC leaves versus empty vector control (left) at 7 days post-infiltration (dpi). **D** qRT-PCR analysis of *BcGUN4.1* transcript levels in overexpression (OE) and control leaves (7 dpi). **E** Pigment content of leaves from *35S:BcGUN4.1* and control at 7 dpi. **F** Phenotypes of *BcGUN4.1*-silenced and control NHCC plants. **G** Relative expression levels of *BcGUN4.1* in leaves of *BcGUN4.1*-silenced and control NHCC plants. **H** Pigment content of leaves of *BcGUN4.1*-silenced and control NHCC plants. Data are presented as means ± standard deviation (SD) of three biological replicates. Statistical significance was determined by Student’s *t*-test (*P < 0.05, **P < 0.01, ***P < 0.001)

Functional characterization through heterologous complementation in *Arabidopsis thaliana*, with transgene expression confirmed by quantitative real-time polymerase chain reaction (qRT-PCR) assays (Fig. S1), produced distinct phenotypic outcomes. The *gun4* mutant exhibited characteristic chlorosis with a reduction in chloroplast number relative to wild-type (WT) plants (Fig. 2A). In contrast, *BcGUN4.1*-complementation lines (*BcGUN4.1*-CL) displayed complete phenotypic rescue, showing darker green coloration and higher chloroplast density than *gun4* mutants (Fig. 2A). Quantitative analysis revealed significantly reduced chlorophyll and carotenoid contents in *gun4* mutants compared with WT. This pigment deficiency was overcome in *BcGUN4.1*-CL lines, with pigment accumulation restored to WT levels (Fig. 2B). These results demonstrate BcGUN4.1’s conserved role in chloroplast development and photosynthetic pigment accumulation.

To validate these findings in the native system, transient overexpression was performed in NHCC. Leaves infiltrated with *35S:BcGUN4.1* exhibited significantly darker green coloration compared with controls after 7 days (Fig. 2C). qRT-PCR analysis revealed 1.8-fold higher *BcGUN4.1* expression in transgenic leaves (Fig. 2D), corresponding to 18.24–58.89% increases in chlorophyll and carotenoid contents (Fig. 2E). Complementary VIGS in NHCC achieved 32–41% reduction in *BcGUN4.1* expression (Fig. 2F–G), leading to 1.70–15.49% decreases in pigment content compared with empty vector controls (Fig. 2H). The agreement between gain- and loss-of-function phenotypes across both heterologous and homologous systems provides strong evidence that *BcGUN4.1* regulates leaf greenness by modulating pigment biosynthesis and chloroplast differentiation in NHCC.

### The BcGUN4.1 protein directly interacts with BcSG1 and BcCHLH

**Fig. 3:**
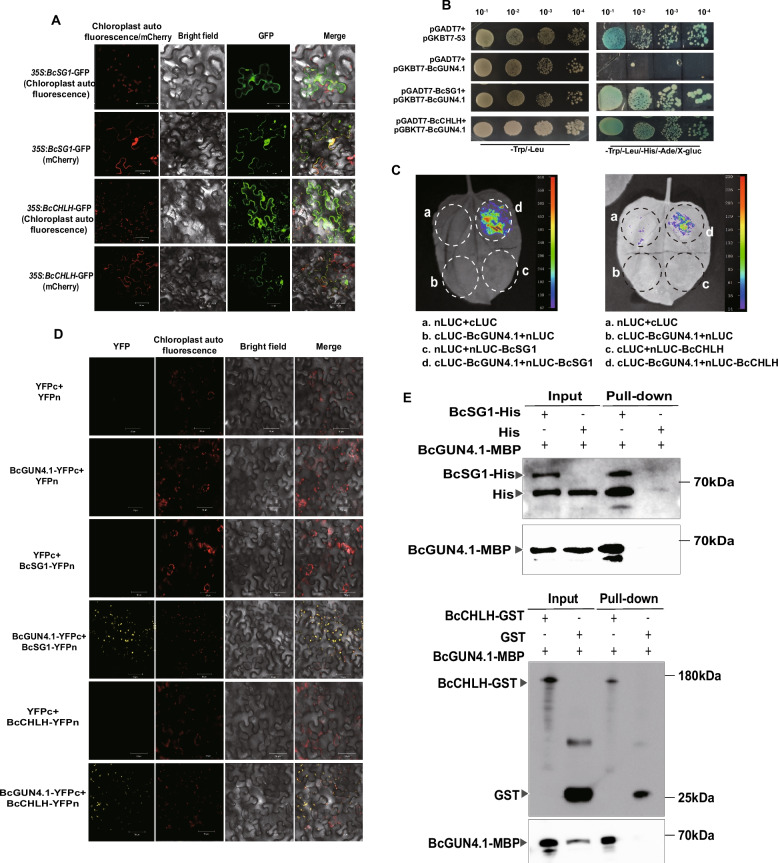
BcGUN4.1 directly interacts with BcSG1 and BcCHLH. **A** Subcellular localization analysis of BcSG1 and BcCHLH. **B** Y2H assay indicates that BcGUN4.1 directly interacts with BcSG1 and BcCHLH. **C** LCI assay confirms that BcGUN4.1 directly interacts with BcSG1 and BcCHLH. **D** BiFC assay indicates that BcGUN4.1 directly interacts with BcSG1 and BcCHLH. **E** Pull-down assay confirms that BcGUN4.1 directly interacts with BcSG1 and BcCHLH in vitro

*BcSG1*, a well-characterized regulator of chloroplast development and leaf pigmentation in NHCC (Hu et al. 2014; Bai et al. 2024), exhibits genetic epistasis with *BcGUN4.1*, as demonstrated in our previous research (Bai et al. 2024). Following the established interaction between CHLH (a catalytic subunit of MgCh) and GUN4 in modulating MgCh activity (Larkin et al. 2003), a detailed analysis of the molecular associations was performed among *BcGUN4.1*, *BcSG1*, and *BcCHLH* in NHCC. Genomic analysis identified both *BcSG1* and *BcCHLH* as single-copy genes in NHCC. Sequence comparison of CHLH orthologs across *A. thaliana* and various *Brassica* species (*B. oleracea*, *B. napus*, *B. rapa* Chiifu, and *B. rapa* NHCC001) revealed high conservation of functional domains (Fig. S2), indicating that BcCHLH maintains evolutionarily preserved roles in chlorophyll biosynthesis. Subcellular localization assays showed that both BcSG1 and BcCHLH localize to chloroplasts and cell membranes (Fig. 3A), similar to BcGUN4.1’s localization pattern, suggesting functional coordination in shared subcellular compartments. To determine physical interactions within this regulatory network, Y2H assays using BcGUN4.1 as bait demonstrated specific interactions with both BcSG1 and BcCHLH prey proteins (Fig. 3B). LCI analysis revealed detectable LUC signals when BcGUN4.1 was co-infiltrated with either BcCHLH or BcSG1, confirming interactions between BcGUN4.1 and both BcCHLH and BcSG1 proteins (Fig. 3C). BiFC analysis validated these interactions within chloroplasts (Fig. 3D). In vitro pull-down assays further confirmed the protein–protein interactions. BcCHLH-glutathione S-transferase (GST) protein formed a complex with BcSG1-hexahistidine (His), BcSG1-His interacted with BcGUN4.1-maltose-binding protein (MBP), while no interaction occurred with GST/His-tagged empty vector controls (Fig. 3E). These results demonstrate that BcGUN4.1 directly interacts with BcSG1 and BcCHLH in vivo and in vitro.

### *BcSG1* positively contributes to pigment accumulation

**Fig. 4:**
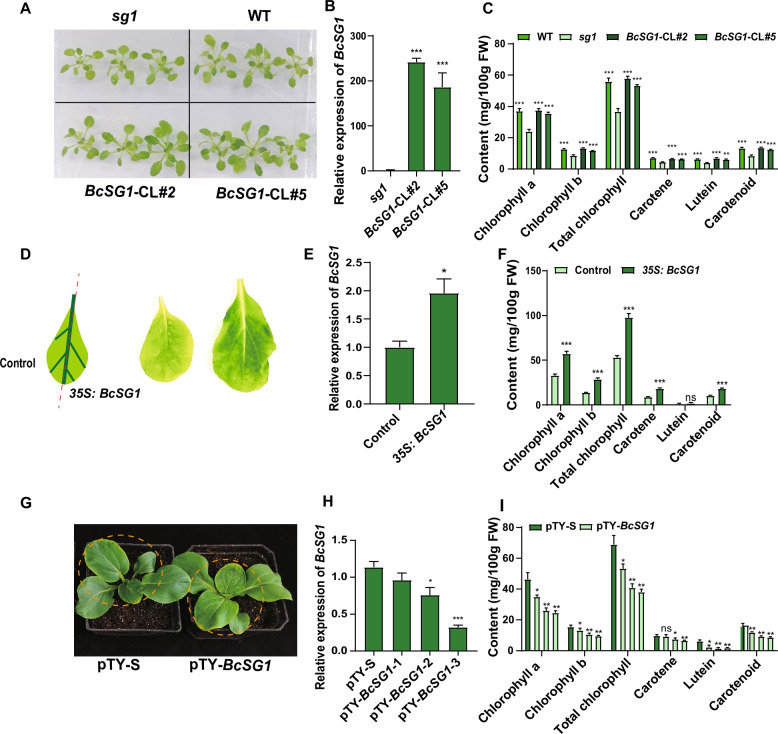
Heterologous complementation, transient overexpression, and VIGS assays reveal that *BcSG1* positively contributes to chlorophyll and carotenoid content. **A** Phenotypes of WT, *sg1* mutants, and complementation lines. **B** Relative expression levels of *BcSG1* in *sg1* mutants and complementation lines. **C** Pigment content comparison among WT, *sg1* mutants, and complementation lines. **D** Phenotypes of leaves transiently overexpressed with *35S:BcSG1* and control. **E** Relative expression levels of *BcSG1* in *35S:BcSG1* and control. **F** Pigment content of leaves of plants transiently overexpressed with *35S:BcSG1* and control. **G** Representative phenotypes of *BcSG1*-silenced and control plants. **H** Relative expression levels of *BcSG1* in *BcSG1*-silenced and control plants. **I** Pigment content comparison between *BcSG1*-silenced and control plants. Data are presented as means ± standard deviation (SD) of three biological replicates. Statistical significance was determined by Student’s *t*-test (*P < 0.05, **P < 0.01, ***P < 0.001)

To validate the function of *BcSG1*, *Arabidopsis sg1* mutants were used for heterologous complementation studies, with complementation lines verified by qRT-PCR. The analysis confirmed successful restoration of *BcSG1* expression in complementation lines (*BcSG1*-CL) to *sg1* levels (Fig. 4A, B). Phenotypic assessment demonstrated that while *sg1* mutants exhibited yellow-green leaves, *BcSG1*-CL showed complete phenotypic restoration with normal green coloration similar to *sg1* (Fig. 4A). Analysis of pigment content revealed significantly higher chlorophyll and carotenoid contents in WT and complementation lines compared with *sg1* mutants (Fig. 4C). These findings were confirmed in NHCC by transient overexpression studies. *35S:BcSG1* lines exhibited 2.1-fold higher *BcSG1* transcript levels (Fig. 4D, E), corresponding to 47–53% increased chlorophyll and carotenoid contents compared with empty vector controls (Fig. 4F). Visual examination confirmed enhanced green pigmentation in overexpression lines (Fig. 4D). VIGS resulted in 55–68% reduction in *BcSG1* expression (Fig. 4G, H), leading to 12–17% decreased chlorophyll and carotenoid contents compared with pTY-S controls (Fig. 4I). This comprehensive validation through gain- and loss-of-function approaches in both heterologous and homologous systems definitively establishes *BcSG1* as a positive regulator of chlorophyll and carotenoid accumulation in NHCC.

### BcGUN4.1, BcSG1, BcCHLH, and BcTPR4 interact with each other

**Fig. 5:**
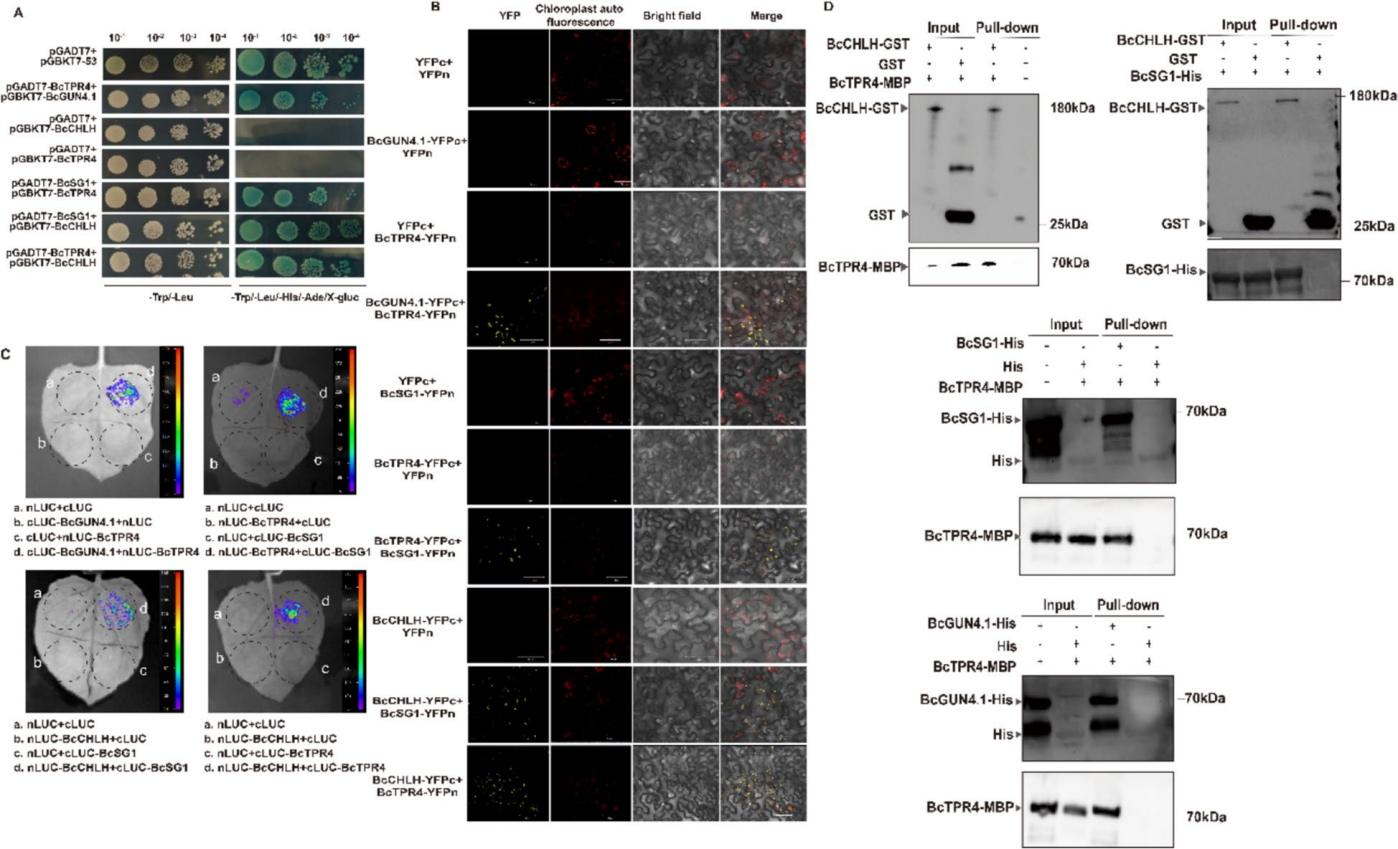
BcGUN4.1, BcSG1, BcCHLH, and BcTPR4 directly interact with each other. **A** Y2H assay indicates that BcGUN4.1, BcSG1, and BcCHLH interact with BcTPR4, and BcCHLH interacts with BcSG1. The protein–protein interactions among BcGUN4.1, BcSG1, BcCHLH, and BcTPR4 were confirmed by BiFC (**B**), LCI (**C**), and pull-down (**D**) assays

Beyond the established protein–protein interactions of BcGUN4.1–BcSG1 and BcGUN4.1–BcCHLH, the complete regulatory network remains to be elucidated. Network analysis predicted that GUN4.1 physically interacts with CHLH, *CHLH* exhibits co-expression with *TPR4*, and TPR4 shares a protein domain with SG1 (Fig. S3). Our research demonstrated the co-localization of BcGUN4.1, BcSG1, and BcCHLH, as well as the interaction capability of BcGUN4.1 with both BcSG1 and BcCHLH (Figs. 1B and 3A). Y2H assays revealed that BcTPR4 interacts with BcGUN4.1, BcSG1, and BcCHLH (Fig. 5A). In addition, a direct interaction was observed between BcCHLH and BcSG1 (Fig. 5A). BiFC assays confirmed these interactions in plants, showing chloroplast-localized fluorescent signals between BcGUN4.1 and BcCHLH, BcGUN4.1 and BcTPR4, BcSG1 and BcCHLH, BcSG1 and BcTPR4, and BcCHLH and BcTPR4 (Fig. 5B). LCI assays provided independent validation of the Y2H and BiFC results (Fig. 5C). In vitro pull-down assays demonstrated that BcCHLH-GST interacted with both BcSG1-His and BcTPR4-MBP, while the GST-tagged empty vector control showed no interaction (Fig. 5D). Similarly, BcSG1-His and BcGUN4.1-His each interacted with BcTPR4-MBP, whereas the His-tagged empty vector control exhibited no binding (Fig. 5D). These systematic interaction patterns suggest the formation of a tetrameric complex comprising BcCHLH, BcSG1, BcTPR4, and BcGUN4.1.

### *BcTPR4* contributes to pigment accumulation

**Fig. 6:**
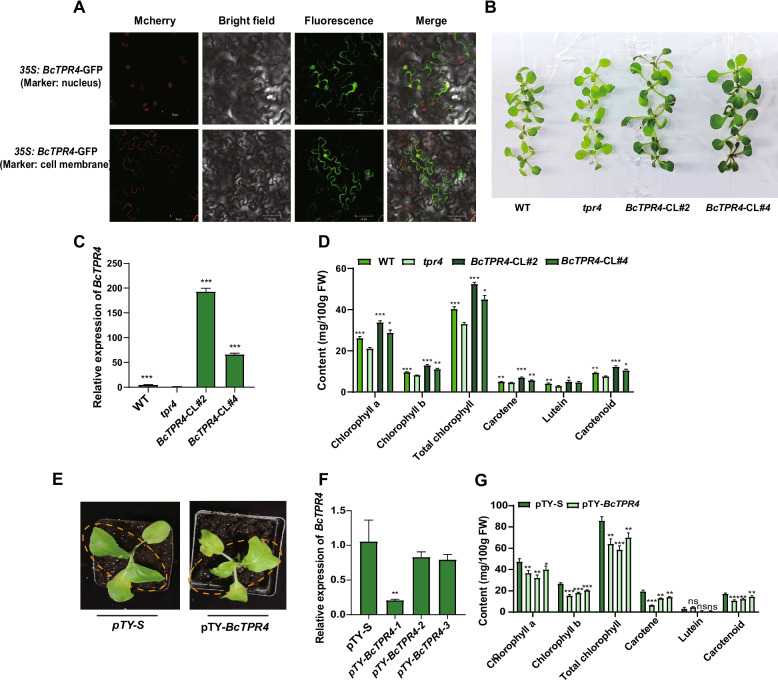
*BcTPR4* contributes to chlorophyll and carotenoid accumulation. **A** Subcellular localization analysis of BcTPR4. **B** Phenotypes of WT, *tpr4*, and complementation lines. **C** Relative expression levels of *BcTPR4* in WT, *tpr4*, and complementation lines. **D** Pigment content of WT, *tpr4*, and complementation lines. **E** Representative phenotypes of pTY-S and *BcTPR4*-silenced plants. **F** Relative expression levels of *BcTPR4* in leaves of *BcTPR4*-silenced and control plants. **G** Pigment content comparison between *BcTPR4*-silenced and control plants. Data are presented as means ± standard deviation (SD) of three biological replicates. Statistical significance was determined by Student’s *t*-test (*P < 0.05, **P < 0.01, ***P < 0.001)

Although TPR4 is characterized as a carboxylate clamp-type TPR protein (Prasad et al. 2010), the role of *BcTPR4* was undefined before this study. To investigate the function of *BcTPR4*, the gene was fused to green fluorescent protein (GFP) under control of the CaMV 35S promoter to determine its subcellular localization. Analysis revealed BcTPR4 localization in the nucleus and cell membranes (Fig. 6A). *Arabidopsis tpr4* mutants were used for heterologous complementation studies. Complementation lines were verified by qRT-PCR, which demonstrated significantly elevated *BcTPR4* expression levels (> 50-fold) compared with *tpr4* mutants (Fig. 6B, C). The *tpr4* mutants exhibited yellow-green leaves and reduced pigment content relative to WT (Fig. 6B, D). Complementation lines displayed dark-green leaves and restored pigment levels (Fig. 6B, D). In addition, VIGS analysis was used to examine *BcTPR4* function in NHCC. Silencing of *BcTPR4* resulted in yellow-green leaves and decreased pigment content, indicating *BcTPR4*’s role in pigment accumulation (Fig. 6E–G). These complementary approaches across heterologous (*Arabidopsis*) and homologous (NHCC) systems provide conclusive evidence that *BcTPR4* functions as a positive regulator of chlorophyll and carotenoid accumulation. The consistent phenotypic effects observed in both experimental systems, coupled with its interaction network with known chlorophyll biosynthesis components, suggest that *BcTPR4* plays a role in the regulation of photosynthetic pigment biosynthesis.

### BcGUN4.1, BcSG1, BcCHLH, and BcTPR4 mutually modulate leaf greenness

**Fig. 7:**
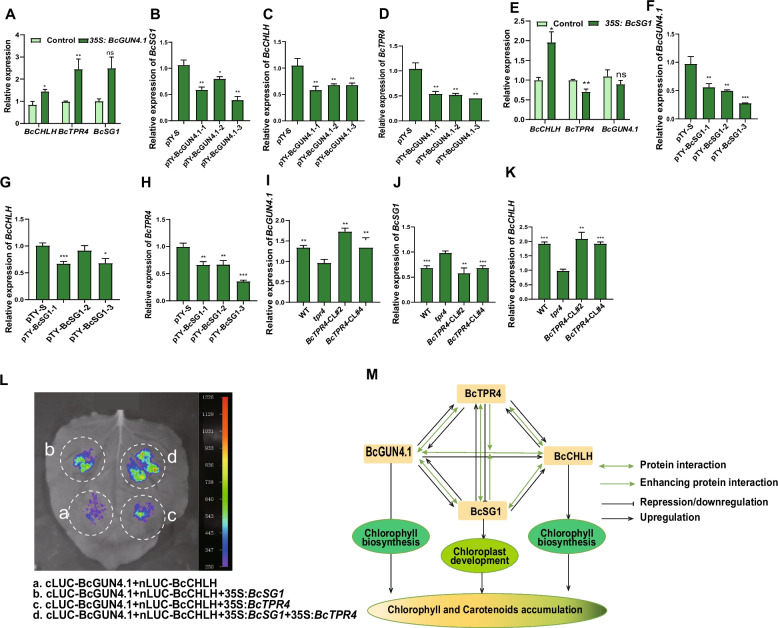
Relationships among BcGUN4.1, BcSG1, BcCHLH, and BcTPR4. **A** Relative expression levels of *BcSG1*, *BcCHLH*, and *BcTPR4* in *35S:BcGUN4.1* and control. **B**–**D** Relative expression levels of *BcSG1*, *BcCHLH*, and *BcTPR4* in *BcGUN4.1*-silenced and control plants. **E** Relative expression levels of *BcGUN4.1*, *BcCHLH*, and *BcTPR4* in *35S:SG1* and control. **F**–**H** Relative expression levels of *BcGUN4.1*, *BcCHLH*, and *BcTPR4* in *BcSG1*-silenced and control plants. **I**–**K** Relative expression levels of *BcGUN4.1*, *BcSG1*, and *BcCHLH* in WT*, tpr4*, and complementation lines. **L** LCI assay indicates that BcSG1 and BcTPR4 could stimulate BcGUN4.1–BcCHLH complex activity. **M** Schematic of the proposed regulatory network among BcGUN4.1, BcSG1, BcCHLH, and BcTPR4. Data are presented as means ± standard deviation (SD) of three biological replicates. Statistical significance was determined by Student’s *t*-test (*P < 0.05, **P < 0.01, ***P < 0.001)

To elucidate the regulatory relationships among *BcGUN4.1*, *BcSG1*, *BcCHLH*, and *BcTPR4*, expression levels were analyzed in overexpressed and silenced lines. *BcGUN4.1* overexpression significantly elevated the expression of *BcSG1*, *BcCHLH*, and *BcTPR4*, with notable increases in *BcSG1* and *BcTPR4* (Fig. 7A). Conversely, BcGUN4.1 silencing decreased the expression of these three genes (Fig. 7B–D), establishing a positive regulatory relationship between *BcGUN4.1* and *BcSG1*, *BcCHLH*, and *BcTPR4*. In contrast, *BcSG1* overexpression enhanced *BcCHLH* expression while moderately suppressing *BcGUN4.1* and *BcTPR4* expression (Fig. 7E). Silencing *BcSG1* decreased the transcript levels of *BcSG1*, *BcCHLH*, and *BcTPR4* (Fig. 7F–H). Furthermore, in *tpr4* complementation lines, *BcGUN4.1* and *BcCHLH* expression increased compared with the mutant, while *BcSG1* expression decreased (Fig. 7I–K), indicating that *BcTPR4* positively regulates *BcGUN4.1* and *BcCHLH* but negatively influences *BcSG1*. These findings demonstrate that these four genes operate in a coordinated regulatory network to modulate pigment accumulation through reciprocal expression regulation.

BcGUN4.1, BcSG1, BcCHLH, and BcTPR4 physically interact to form a functional tetrameric complex (Figs. 3 and 5), suggesting cooperative regulation of pigment accumulation. This observation aligns with previous studies showing GUN4–CHLH interactions are essential for chlorophyll biosynthesis through MgCh activation (Adhikari et al. 2011). GUN4 enhances MgCh activity and facilitates CHLH’s association with chloroplast membranes (Adhikari et al. 2011), supporting the hypothesis that the BcGUN4.1–BcCHLH complex functions as the core module within the tetramer. A split-LUC assay, where BcGUN4.1 and BcCHLH were fused to N- and C-terminal LUC fragments, revealed that co-infiltration with either *35S:BcSG1* or *35S:BcTPR4* significantly enhanced the LUC signal compared with controls (Fig. 7L). Notably, simultaneous infiltration with both *35S:BcSG1* and *35S:BcTPR4* generated the strongest luminescence signal (Fig. 7L), demonstrating synergistic enhancement of BcGUN4.1–BcCHLH complex activity. These results establish BcSG1 and BcTPR4 as regulatory subunits that enhance the core BcGUN4.1–BcCHLH complex, with optimal activation occurring when both subunits are present (Fig. 7M). This molecular mechanism explains their collective role in promoting pigment accumulation.

## Discussion

Leaf coloration constitutes an essential agronomic trait in leafy vegetable breeding, primarily regulated by the biosynthesis and accumulation of endogenous pigments (Amagai et al. 2022). Anthocyanins contribute to purple, red, and pink colorations, while chlorophylls and carotenoids determine green, yellow, and orange hues (Li et al. 2017; Zhang et al., 2022; Zhao et al. 2022). Green pigmentation remains predominant in the NHCC germplasm. This study identified three key genes (*BcGUN4.1*, *BcSG1*, and *BcTPR4*) that function as positive regulators of pigment accumulation (Figs. 2, 4, and 6).

Tetrapyrrole biosynthesis functions as the central pathway for chlorophyll and heme production in plants (Foelsche et al. 2022). GUN4, a key component of retrograde signaling, enhances tetrapyrrole biosynthesis by binding Proto and MgProto while interacting with CHLH (Larkin et al. 2003; Adhikari et al. 2011). This investigation identified two *BcGUN4* genes in NHCC, with *BcGUN4.1* demonstrating a specific correlation with leaf greenness (Fig. 2). Functional analyses through transient overexpression and VIGS assays in NHCC and heterologous complementation in *Arabidopsis* definitively establish *BcGUN4.1* as a positive regulator of pigment accumulation (Fig. 2). These findings position *BcGUN4.1* as a critical determinant of leaf greenness in NHCC. Related studies have documented that reduced *GUN4* expression leads to leaf yellowing (Xie et al. 2019). *GUN4* plays an essential role in rice chlorophyll biosynthesis, with mutants exhibiting albino phenotypes (Zhou et al. 2012). In *Arabidopsis*, chlorophyll content was reduced in *gun4* mutants but stable in overexpression lines (Peter & Grimm 2009). While *Arabidopsis* GUN4 is exclusively localized to chloroplasts (Li et al. 2023), BcGUN4.1 exhibits dual localization in both chloroplasts and cell membranes (Fig. 1B), indicating potential functions in membrane-associated signaling. The distinct subcellular distribution of BcGUN4.1 implies its enhanced capacity as a potential receptor or transporter in nucleus-to-chloroplast communication.

BcSG1 and BcTPR4 are members of the TPR-containing protein family, which plays essential roles in thylakoid membrane biogenesis and chlorophyll biosynthesis (Stockel et al. 2006; Zhang et al. 2015; Bohne et al. 2016). *BcSG1* has been identified as a critical determinant of leaf greenness in NHCC (Bai et al. 2024). The present study demonstrates that *BcSG1* positively regulates pigment accumulation (Fig. 4), aligning with findings in *Arabidopsis*, where SG1 has been identified as a chloroplast-localized protein (Hu et al. 2014). Notably, BcSG1 exhibits dual localization to chloroplasts and cell membranes (Fig. 3A), indicating potential roles in interorganellar signaling beyond its established chloroplast functions. Similarly, *BcTPR4* promotes pigment accumulation (Fig. 6). BcTPR4 appears to regulate pigment accumulation through two mechanisms: (1) transcriptional regulation of chlorophyll biosynthesis and chloroplast development-related genes (*BcGUN4.1*, *BcCHLH*, and *BcSG1*), and (2) direct protein interactions with BcGUN4.1, BcSG1, and BcCHLH proteins to enhance their enzyme activities. The precise molecular mechanisms warrant further investigation. Although BcTPR4 primarily localizes to the nucleus and cell membranes, it exhibits functional interactions with chloroplast/membrane-localized proteins (BcGUN4.1, BcSG1, and BcCHLH) in chloroplasts. This observation may be explained by conditional chloroplast targeting through non-canonical transit peptides or post-translational modifications (e.g., phosphorylation) and membrane contact site-mediated long-distance interactions between organelles. The specific mechanisms underlying this cross-compartmental regulation require further examination in subsequent studies.

The results demonstrate that *BcGUN4.1*, *BcSG1*, and *BcTPR4* mutually regulate each other’s expression levels (Fig. 7). As established components of chloroplast development (Hu et al. 2014), changes in *BcGUN4.1* or *BcSG1* expression (by overexpression or silencing) substantially affect chloroplast abundance and subsequently modify the expression of pigment biosynthesis-related genes. *BcSG1* overexpression significantly elevated BcCHLH expression, while reducing the expression levels of *BcGUN4.1* and *BcTPR4*. In contrast, silencing of *BcSG1* resulted in a significant decrease in the expression levels of *BcCHLH*, *BcGUN4.1*, and *BcTPR4* (Fig. 7). *BcSG1* functions as a positive regulator of *BcCHLH* expression. The reduced expression of *BcGUN4.1* and *BcTPR4* upon *BcSG1* silencing indicates that their basal expression depends on BcSG1 presence or activity. However, increased levels of *BcSG1* exhibit an inhibitory effect on *BcGUN4.1* and *BcTPR4* expression. This represents a dose-dependent regulatory mechanism, where *BcSG1* is essential for basal expression but acts as a repressor above a certain threshold. In addition, the expression patterns of *BcGUN4.1* and *BcTPR4* exhibited consistent co-downregulation under both overexpression and silencing conditions of *BcSG1*, suggesting their regulation by similar mechanisms. The underlying mechanism merits further investigation. Notably, increased *BcTPR4* expression enhanced *BcGUN4.1* and *BcCHLH* expression while suppressing *BcSG1* expression (Fig. 7I–K), indicating potential functional competition between these TPR-containing proteins. This reciprocal regulation suggests a negative feedback loop between *BcTPR4* and *BcSG1*, where *BcTPR4* suppression increases *BcSG1* expression, a mechanism requiring further research. Multiple assays revealed that these four proteins form a functional tetrameric complex (Fig. 5). The core BcGUN4.1–BcCHLH module, essential for chlorophyll biosynthesis (Larkin et al. 2003; Adhikari et al. 2011), demonstrates enhanced activity when associated with BcSG1, BcTPR4, or both (Fig. 7L). Maximum stimulation occurs with both TPR-containing proteins present, indicating their synergistic role in optimizing complex functionality. This suggests that BcSG1 and BcTPR4 contribute to chlorophyll biosynthesis by transcriptional regulation and direct enhancement of BcGUN4.1–BcCHLH enzyme activity. The TPR domains in BcSG1 and BcTPR4 facilitate these functions by mediating protein–protein interactions (Das et al. 1998; Blatch and Lässle 1999) and maintaining complex stability (Tseng et al. 2001). These proteins likely serve as regulatory scaffolds by enhancing BcGUN4.1–BcCHLH complex stability, potentially recruiting additional biosynthetic components, and regulating chlorophyll production efficiency (Fig. 7M). This tetrameric complex represents a key determinant of intense leaf greenness in NHCC varieties. While specific mechanisms require further investigation, these findings provide important insights into the genetic basis of leaf coloration and offer molecular targets for breeding programs focused on optimizing vegetative pigmentation.

In summary, the results demonstrate that BcGUN4.1, BcSG1, BcCHLH, and BcTPR4 regulate pigment accumulation through both independent and coordinated mechanisms (Fig. 7M). These proteins form an interaction network that synergistically modulates leaf greenness in NHCC, with their physical interactions and regulatory relationships establishing a sophisticated system for chlorophyll biosynthesis and chloroplast development (Fig. 7M).

## Materials and methods

### Plant material and growth conditions

The three NHCC varieties (‘Suzhouqing’, ‘49caixin’, and ‘Wutacai’), *A. thaliana*, and tobacco (*Nicotiana benthamiana*) were sown directly and cultivated in a growth chamber under the following conditions: 150 μmol∙m^−2^∙s^−1^ light intensity, 55% relative humidity, and 16/8 light/dark cycles at 22 °C (dark) and 25 °C (light). The *gun4* (SALK_016395C), *sg1* (SALK_046229C), and *tpr4* (SALK_108627C) mutants were obtained from AraShare.

### Pigment extraction and measurement

Leaf samples (approximately 0.1 g) were collected and frozen in liquid nitrogen for subsequent analysis. Pigment content was measured following the method described by Xu et al. (2013) with minor modifications. Pigments were extracted using 1 mL of extraction mixture (ethanol:acetone = 1:1) under dark conditions at 25 °C for 24 h. The supernatant was analyzed using a multi-detection microplate reader (Cytation 3; BioTek, Winooski, VT, USA) at wavelengths of 470 nm, 474 nm, 485 nm, 642 nm, 649 nm, and 665 nm. Pigment content was measured according to the methods outlined by Bai et al. (2024).

### Identification of *BcGUN4* and multiple sequence alignment

The amino acid sequences of BcGUN4.1 and BcGUN4.2 were obtained from the Non-heading Chinese Cabbage and Watercress Database (NCCWDB) (Li et al. 2020). BLASTp analysis was used to identify BrGUN4 in *B. rapa* Chiifu and AtGUN4 with an e-value of 1e − 5. The amino acid sequences of CHLH in *A. thaliana*, *B. rapa* Chiifu, *B. napus*, *B. oleracea*, and *B. rapa* NHCC001 were retrieved from the Brassicaceae Database (http://www.brassicadb.cn/#/) and NCCWDB. Multiple sequence alignment was performed using Multiple Alignment, and the results were visualized using ESPript v3.0.

### Subcellular localization assay

The CDSs of *BcGUN4.1*, *BcSG1*, *BcCHLH*, and *BcTPR4* were cloned from ‘Wutacai’ cDNA. The CDS lacking the stop codon was inserted into the pRI101-GFP vector containing the CaMV 35S promoter using *Nde*I and *Bam*HI restriction sites through homologous recombination. The primers used for cloning and subcloning are listed in Table S1. The recombinant plasmid and empty vector were transformed into *Agrobacterium tumefaciens* strain GV3101 for tobacco leaf infiltration as described by Li et al. (2022). Infiltrated tobacco plants were incubated in dark conditions at room temperature for 12 h before transfer to normal growth conditions for 72 h. Fluorescence was observed using a confocal laser scanning microscope (LSM780; Zeiss, Jena, Germany).

### Transient genetic transformation in the NHCC

The *Agrobacterium* mixture containing *35S:BcGUN4.1*, *35S:BcSG1*, and *35S:GFP* was maintained at OD_600_ = 0.2. ‘49caixin’ plants with two young leaves were selected for infiltration. Each leaf was infiltrated with *35S:BcGUN4.1*/*35S:BcSG1* on one half, while the other half functioned as a control. After 7 days, leaves were examined and sampled for pigment content measurement and RNA extraction. The experiment was performed in triplicate, with 20 leaves infiltrated per biological replicate.

### Genetic transformation of *Arabidopsis thaliana*

Genetic transformation was performed using *Agrobacterium* containing *35S:BcGUN4.1*/*35S:BcSG1*/*35S:BcTPR4*. *gun4*, *sg1*, and *tpr4* were used for transformation. The *Agrobacterium* solution was maintained at OD_600_ = 0.8. Transgenic *A. thaliana* plants were generated using the *Agrobacterium*-mediated floral dip method as described by Li et al. (2022). Transgenic lines were selected on media containing 50 mg∙L^−1^ kanamycin for three generations. The transgenic plants were verified using qRT-PCR.

### Virus-induced gene silencing (VIGS) in the NHCC

The 80-bp fragments were designed for *BcGUN4.1*, *BcSG1*, and *BcTPR4*, which were synthesized and subcloned into the *pTY* vector by GenScript Company (Nanjing, China) (Yu et al. 2019). The fragment sequences are listed in Table S2. *pTY-BcGUN4.1*, *pTY-BcSG1*, *pTY-BcTPR4*, and *pTY* vectors were introduced into two-week-old ‘Suzhouqing’ plant leaves through gene-gun-mediated transformation (1300 psi, PDS-1000/He; Bio-Rad Laboratories, Hercules, CA, USA). After one month, silencing efficiency was evaluated using qRT-PCR.

### RNA extraction and qRT-PCR

Total RNA was extracted using the *SteadyPure* Plant RNA Extraction Kit (Accurate Biology, Changsha, China). Reverse transcription was performed using the HiScript IV 1 st Strand cDNA Synthesis Kit (+ gDNA wiper) (Vazyme, Nanjing, China). The SYBR Green Premix *Pro Taq* HS qPCR Kit (Low ROX Plus) (Accurate Biology, Changsha, China) was used for qRT-PCR in a thermal cycler (iQ5; Bio-Rad Laboratories). The PCR procedure (95 °C for 30 s, 95 °C for 5 s, 60 °C for 30 s, 40 cycles) followed Yu et al. (2024). *BcActin7* and *AtActin* functioned as internal references for qRT-PCR in NHCC and *Arabidopsis*, respectively. The 2^−∆∆Ct^ method was used to calculate the relative expression (Pfaffl 2001). Each sample was analyzed with three biological replicates and three technical replicates. The relevant primers are listed in Table S1.

### Y2H assay

The CDSs of *BcSG1*, *BcCHLH*, and *BcTPR4* were subcloned and inserted into pGADT7 between the *Eco*RI and *Bam*HI restriction sites. The CDSs of *BcGUN4.1*, *BcCHLH*, and *BcTPR4* were inserted into the pGBKT7 vector between the *Eco*RI and *Bam*HI restriction sites. The combinations of BcGUN4.1-pGBKT7 and pGADT7, BcGUN4.1-pGBKT7 and BcSG1-pGADT7, BcGUN4.1-pGADT7 and BcCHLH-pGBKT7, BcGUN4.1-pGBKT7 and BcTPR4-pGADT7, BcCHLH-pGBKT7 and BcSG1-pGADT7, BcCHLH-pGBKT7 and BcTPR4-pGADT7, BcCHLH-pGBKT7 and pGADT7, BcTPR4-pGBKT7 and pGADT7, and BcTPR4-pGBKT7 and BcSG1-pGADT7 were co-transformed into Y2H Gold yeast. The transformed yeast cells were cultured on SD/− Trp/− Leu media at 28 °C for 4 days and subsequently transferred to SD/− Trp/− Leu/− His/− Ade media supplemented with 5-bromo-4-chloro-3-indolyl β-D-glucuronide cyclohexylammonium salt for protein interaction screening. The relevant primers are listed in Table S1.

### BiFC assay

The CDSs of *BcGUN4.1*, *BcCHLH*, and *BcTPR4* were subcloned and inserted into the pFGC5941-C-YFP vector, with the proteins fused to the C-terminus of YFP. The BcSG1, BcCHLH, and BcTPR4 proteins were fused with the N-terminus of YFP. All recombinant plasmids were transformed into *Agrobacterium* GV3101 cells for infiltration. Four-week-old tobacco blades were inoculated with the following combinations: BcGUN4.1-YFPc × BcSG1-YFPn/BcCHLH-YFPn/BcTPR4-YFPn, BcCHLH-YFPc × BcSG1-YFPn/BcTPR4-YFPn, and BcTPR4-YFPc × BcSG1-YFPn. After 72 h, fluorescence signals were detected using a confocal laser scanning microscope (LSM780; Zeiss, Jena, Germany).

### LCI assay

The CDSs of *BcSG1*, *BcCHLH*, and *BcTPR4* were subcloned into the pCAMBIA1300-nLUC vector, while the CDSs of *BcGUN4.1*, *BcSG1*, *BcCHLH*, and *BcTPR4* were subcloned into the pCAMBIA1300-cLUC vector. The recombinant plasmids were transformed into *Agrobacterium* GV3101 cells. The following combinations (BcGUN4.1-cLUC × BcCHLH-nLUC/BcSG1-nLUC/BcTPR4-nLUC, BcSG1-cLUC × BcCHLH-nLUC/BcTPR4-nLUC, and BcTPR4-cLUC × BcCHLH-nLUC) were co-infiltrated into *N. benthamiana* leaves to identify protein–protein interactions. Fluorescence signals were detected 72 h after infiltration using a live imaging system (Tanon 4600; Tanon Science & Technology, Shanghai, China).

### Pull-down assay

The CDS of *BcCHLH* was inserted into the pGEX-4 T-1 vector to express the GST fusion protein BcCHLH-GST. The CDSs of *BcSG1* and *BcGUN4.1* were inserted into the pCold-TF vector to express His-tagged fusion proteins (BcSG1-His and BcTPR4-His). The CDSs of *BcGUN4.1* and *BcTPR4* were inserted into the pMAL-c2 × vector (New England Biolabs, Ipswich, MA, USA) to express MBP fusion proteins (BcGUN4.1-MBP and BcTPR4-MBP). The recombinant plasmids were transformed into *Escherichia coli* BL21(DE3) competent cells. Protein expression was induced with 1 mM isopropyl β-D-1-thiogalactopyranoside at 16 °C for 16 h. BcCHLH-GST and the GST control protein were purified using a GST-Tag Protein Purification Kit with Magnetic Agarose Beads (Beyotime, Shanghai, China). BcSG1-His, BcGUN4.1-His, and the His control protein were purified using a His-Tag Protein Purification Kit (Beyotime). BcGUN4.1-MBP and BcTPR4-MBP were purified using a PurKine™ MBP-Tag Protein Purification Kit (Dextrin) (Abbkine, Wuhan, China). Pull-down assays were performed using the following combinations: BcCHLH-GST/GST × BcSG1-His/BcGUN4.1-MBP/BcTPR4-MBP, BcSG1-His/His × BcTPR4-MBP/BcGUN4.1-MBP, and BcGUN4.1-His/His × BcTPR4-MBP. Protein interactions were analyzed by Western blotting.

### Statistical analysis

Statistical significance was determined using *t*-tests, with *P < 0.05, **P < 0.01, and ***P < 0.001. Graphs were generated using GraphPad Prism 8 (GraphPad Software, La Jolla, CA, USA).

## Supplementary Information

Supplementary Material 1: Fig. S1. Relative expression levels of BcGUN4.1 in WT, *gun4*, and complementary lines.

Supplementary Material 2: Fig. S2. The multiple amino acid sequence alignment of CHLH in *B. oleracea*, *B. napus*, *B. rapa* Chiifu, and *B. rapa* NHCC001.

Supplementary Material 3: Fig. S3. Regulatory network between SG1 and GUN4 predicted via GeneMANIA.

Supplementary Material 4: Table S1. Primes for qRT-PCR analysis and fragment amplification. Table S2. Sequences of 80-bp fragments of BcGUN4.1, BcSG1, and BcTPR4, were designed for the VIGS assay.

## Data Availability

The data are presented within the paper and supplementary files.

## Abbreviations

GUN4: GENOME UNCOUPLE 4
SG1: SLOW GREEN 1
VIGS: Virus-induced gene silencing
Y2H: Yeast two hybrid
BiFC: Bimolecular fluorescence complementation
LCI: Firefly luciferase complementation imaging
