# Supplementary figures and images for: Convergent roles of BcGUN4.1, BcSG1, BcCHLH, and BcTPR4 in regulating the leaf greenness of non-heading Chinese cabbage
Source: Mol Hortic. 2026 May 8;6:32. doi: 10.1186/s43897-025-00216-5 (PMC13154631; doi:10.1186/s43897-025-00216-5)

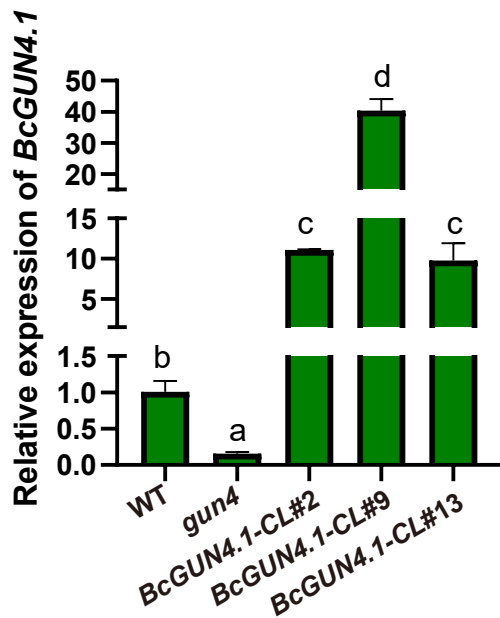

Fig. S1 The relative expression level of *BcGUN4.1* in WT, *gun4*, and complementary lines

Supplement: Supplementary file 1 — Supplementary Material 1: Fig. S1. Relative expression levels of BcGUN4.1 in WT, gun4, and complementary lines. [file 43897_2025_216_MOESM1_ESM.pdf]
