# Supplementary material for: Convergent roles of BcGUN4.1, BcSG1, BcCHLH, and BcTPR4 in regulating the leaf greenness of non-heading Chinese cabbage
Source: Mol Hortic. 2026 May 8;6:32. doi: 10.1186/s43897-025-00216-5 (PMC13154631; doi:10.1186/s43897-025-00216-5)
Supplement: Supplementary file 2 — Supplementary Material 2: Fig. S2. The multiple amino acid sequence alignment of CHLH in B. oleracea, B. napus, B. rapa Chiifu, and B. rapa NHCC001. [file 43897_2025_216_MOESM2_ESM.pdf]

```
           1         10        20        30        40        50
BrCHLH   .MASSLMYSPFTLSTSKAEHLSSLSNTTSKHSFLRRKS.FSKPTNSLFKVKSAVSGNGLFT
BcCHLH   .MASSLMYSPFTLSTSKAEHLSSLSNTTSKHSFLRRKS.FSKPTNSLFKVKSAVSGNGLFT
BoCHLH   MASSLMYSPFTLSASRAEHLSSLSNTTTKHSFLRRKSKPTKPATSIFKVKSSVSGNGLFT
BnaCHLH  MASSLMYSPFTLSASRAEHLSSLSNTTTKHSFLRRKSKPTKPATSIFKVKSSVSGNGLFT
AtCHLH   .MASLVYSPFTLSTSKAEHLSSLTN.STKHSFLRKKHRSTKPAKSFFKVKSAVSGNGLFT

         60        70        80        90        100       110
BrCHLH   QTNPEVRRIVPVKRDNVPTVKIVYVVLEAQYQSSLSEAVQQLNKTSRFASYEVVGYLVEE
BcCHLH   QTNPEVRRIVPVKRDNVPTVKIVYVVLEAQYQSSLSEAVQQLNKTSRFASYEVVGYLVEE
BoCHLH   QTNPEVRRIVPVKRDNVPTVKIVYVVLEAQYQSSLSEAVQQLNKTSRFASYEVVGYLVEE
BnaCHLH  QTNPEVRRIVPVKRDNVPTVKIVYVVLEAQYQSSLSEAVQQLNKTSRFASYEVVGYLVEE
AtCHLH   QTNPEVRRIVPIKRDNVPTVKIVYVVLEAQYQSSLSEAVQSLNKTSRFASYEVVGYLVEE

         120       130       140       150       160       170
BrCHLH   LRDKNTYKSFCKDLEDANIFIGSLIFVEELALKVKEAVEKERDRMDAVLVFPSMPEVMRL
BcCHLH   LRDKNTYKSFCKDLEDANIFIGSLIFVEELALKVKDAVEKERDRMDAVLVFPSMPEVMRL
BoCHLH   LRDKNTYKSFCKDLEDANIFIGSLIFVEELALKVKDAVEKERDRMDAVLVFPSMPEVMRL
BnaCHLH  LRDKNTYKSFCKDLEDANIFIGSLIFVEELALKVKDAVEKERDRMDAVLVFPSMPEVMRL
AtCHLH   LRDKNTYNNFCEDLKDANIFIGSLIFVEELAIKVKDAVEKERDRMDAVLVFPSMPEVMRL

         180       190       200       210       220       230
BrCHLH   NKLGSFSMSQLGQSKSPFFQLFKRKKGAGSAGFADSMLKLVRTLPKVLKYLPSDKAQDAR
BcCHLH   NKLGSFSMSQLGQSKSPFFQLFKRKKGAGSAGFADSMLKLVRTLPKVLKYLPSDKAQDAR
BoCHLH   NKLGSFSMSQLGQSKSPFFQLFKRKKGAGSAGFADSMLKLVRTLPKVLKYLPSDKAQDAR
BnaCHLH  NKLGSFSMSQLGQSKSPFFQLFKRKKGAGSAGFADSMLKLVRTLPKVLKYLPSDKAQDAR
AtCHLH   NKLGSFSMSQLGQSKSPFFQLFKRKK.QGSAGFADSMLKLVRTLPKVLKYLPSDKAQDAR

         240       250       260       270       280       290
BrCHLH   LYILSLQFWLGGSPDNLQNFVKMISGSYIPALKGVKIEYSDPVLFLDTGIWHPLAPTMYD
BcCHLH   LYILSLQFWLGGSPDNLQNFVKMISGSYIPALKGVKIEYSDPVLFLDTGIWHPLAPTMYD
BoCHLH   LYILSLQFWLGGSPDNLQNFVKMISGSYIPALKGVKIEYSDPVLFLDTGIWHPLAPTMYD
BnaCHLH  LYILSLQFWLGGSPDNLQNFVKMISGSYIPALKGVKIEYSDPVLFLDTGIWHPLAPTMYD
AtCHLH   LYILSLQFWLGGSPDNLQNFVKMISGSYVPALKGVKIEYSDPVLFLDTGIWHPLAPTMYD

         300       310       320       330       340       350
BrCHLH   DVKEYLNWYDTRRDTNASLKRKDATVIGLVLQRSHIVTGDDSHYVAVIMELEARGAKVIP
BcCHLH   DVKEYLNWYDTRRDTNASLKRKDATVIGLVLQRSHIVTGDDSHYVAVIMELEARGAKVIP
BoCHLH   DVKEYLNWYDTRRDTNASLKRKDATVIGLVLQRSHIVTGDDSHYVAVIMELEARGAKVIP
BnaCHLH  DVKEYLNWYDTRRDTNASLKRKDATVIGLVLQRSHIVTGDDSHYVAVIMELEARGAKVIP
AtCHLH   DVKEYWNWYDTRRDTNDSLKRKDATVVGLVLQRSHIVTGDDSHYVAVIMELEARGAKVVP

         360       370       380       390       400       410
BrCHLH   IFAGGLDFSGPVERYFVDPVTKQPIINSAVSLTGFALVGGPARQDHPRAIEALKTLDVPY
BcCHLH   IFAGGLDFSGPVERYFVDPVTKQPIINSAVSLTGFALVGGPARQDHPRAIEALKTLDVPY
BoCHLH   IFAGGLDFSGPVERYFVDPVTKQPIINSAVSLTGFALVGGPARQDHPRAIEALKTLDVPY
BnaCHLH  IFAGGLDFSGPVERYFVDPVTKQPIINSAVSLTGFALVGGPARQDHPRAIEALKTLDVPY
AtCHLH   IFAGGLDFSGPVEKYFVDPVSKQPIVNSAVSLTGFALVGGPARQDHPRAIEALKKLDVPY

         420       430       440       450       460       470
BrCHLH   LVGVPLVFQTTEEWLNSTLGLHPIQVALQVALPELDGGMEPIVFAGRDPRTGKSHALHKR
BcCHLH   LVGVPLVFQTTEEWLNSTLGLHPIQVALQVALPELDGGMEPIVFAGRDPRTGKSHALHKR
BoCHLH   LVGVPLVFQTTEEWLNSTLGLHPIQVALQVALPELDGGMEPIVFAGRDPRTGKSHALHKR
BnaCHLH  LVGVPLVFQTTEEWLNSTLGLHPIQVALQVALPELDGGMEPIVFAGRDPRTGKSHALHKR
AtCHLH   LVAVPLVFQTTEEWLNSTLGLHPIQVALQVALPELDGAMEPIVFAGRDPRTGKSHALHKR

         480       490       500       510       520       530
BrCHLH   VEQLCIRAIRWGELKRKTKAEKRVAITVFSFPPDKGNVGTAAYLNVFASIYSVLKDLKRD
BcCHLH   VEQLCIRAIRWGELKRKTKAEKRVAITVFSFPPDKGNVGTAAYLNVFASIYSVLKDLKRD
BoCHLH   VEQLCIRAIRWGELKRKTKAEKRVAITVFSFPPDKGNVGTAAYLNVFASIYSVLKDLKRD
BnaCHLH  VEQLCIRAIRWGELKRKTKAEKRVAITVFSFPPDKGNVGTAAYLNVFASIYSVLKDLKRD
AtCHLH   VEQLCIRAIRWGELKRKTKAEKKLAITVFSFPPDKGNVGTAAYLNVFASIFSVLRDLKRD
```

```
          540       550       560       570       580       590
BrCHLH    GYNVEGLPETAETLIEEILHDKEAQFSSPNLNVAYKMGVREYQSLTPYAAALEENWGKPP
BcCHLH    GYNVEGLPETAETLIEEILHDKEAQFSSPNLNVAYKMGVREYQSLTPYAAALEENWGKPP
BoCHLH    GYNVEGLPETAETLIEEILHDKEAQFSSPNLNVAYKMGVREYQSLTPYAAALEENWGKPP
BnaCHLH   GYNVEGLPETAETLIEEILHDKEAQFSSPNLNVAYKMGVREYQSLTPYAAALEENWGKPP
AtCHLH    GYNVEGLPENAETLIEEIIHDKEAQFSSPNLNVAYKMGVREYQDLTPYANALEENWGKPP

          600       610       620       630       640       650
BrCHLH    GNLNSDGENLLVFGKTYGNVFIGVQPTFGYEGDPMRLLFSKSASPHHGFAAYYSYVEKIF
BcCHLH    GNLNSDGENLLVFGKTYGNVFIGVQPTFGYEGDPMRLLFSKSASPHHGFAAYYSYVEKIF
BoCHLH    GNLNSDGENLLVFGKTYGNVFIGVQPTFGYEGDPMRLLFSKSASPHHGFAAYYSYVEKIF
BnaCHLH   GNLNSDGENLLVFGKTYGNVFIGVQPTFGYEGDPMRLLFSKSASPHHGFAAYYSYVEKIF
AtCHLH    GNLNSDGENLLVYGKAYGNVFIGVQPTFGYEGDPMRLLFSKSASPHHGFAAYYSYVEKIF

          660       670       680       690       700       710
BrCHLH    KADAVLHFGTHGSLEFMPGKQVGMSDACFPDSLIGNIPNVYYYAANNPSEATIAKRRSYA
BcCHLH    KADAVLHFGTHGSLEFMPGKQVGMSDACFPDSLIGNIPNVYYYAANNPSEATIAKRRSYA
BoCHLH    KADAVLHFGTHGSLEFMPGKQVGMSDACFPDSLIGNIPNVYYYAANNPSEATIAKRRSYA
BnaCHLH   KADAVLHFGTHGSLEFMPGKQVGMSDACFPDSLIGNIPNVYYYAANNPSEATIAKRRSYA
AtCHLH    KADAVLHFGTHGSLEFMPGKQVGMSDACFPDSLIGNIPNVYYYAANNPSEATIAKRRSYA

          720       730       740       750       760       770
BrCHLH    NTISYLTPPAENAGLYKGLKQLSELISSYQSLKDTGRGPQIVSSIISTAKQCNLDKDVDL
BcCHLH    NTISYLTPPAENAGLYKGLKQLSELISSYQSLKDTGRGPQIVSSIISTAKQCNLDKDVDL
BoCHLH    NTISYLTPPAENAGLYKGLKQLSELISSYQSLKDTGRGPQIVSSIISTAKQCNLDKDVDL
BnaCHLH   NTISYLTPPAENAGLYKGLKQLSELISSYQSLKDTGRGPQIVSSIISTAKQCNLDKDVDL
AtCHLH    NTISYLTPPAENAGLYKGLKQLSELISSYQSLKDTGRGPQIVSSIISTAKQCNLDKDVDL

          780       790       800       810       820       830
BrCHLH    PDEGTDLSVKERDLVVGKVYSKIMEIESRLLPCGLHVIGEPPSAMEAVATLVNIAALDRA
BcCHLH    PDEGTDLSVKERDLVVGKVYSKIMEIESRLLPCGLHVIGEPPSAMEAVATLVNIAALDRA
BoCHLH    PEEGTDLSVKERDLVVGKVYSKIMEIESRLLPCGLHVIGEPPSAMEAVATLVNIAALDRA
BnaCHLH   PEEGTDLSVKERDLVVGKVYSKIMEIESRLLPCGLHVIGEPPSAMEAVATLVNIAALDRA
AtCHLH    PDEGLELSPKDRDSVVGKVYSKIMEIESRLLPCGLHVIGEPPSAMEAVATLVNIAALDRP

          840       850       860       870       880       890
BrCHLH    EEEISSLPSILAECVGRQIEDVYRGSDKGILSDVELLKQITDASRGAVSAFVEKTTNDKG
BcCHLH    EEEISSLPSILAECVGRQIEDVYRGSDKGILSDVELLKQITDASRGAVSAFVEKTTNDKG
BoCHLH    EEEISSLPSILAECVGRQIEDVYRGSDKGILSDVELLKQITDASRGAVSAFVEKTTNSKG
BnaCHLH   EEEISSLPSILAECVGRQIEDVYRGSDKGILSDVELLKQITDASRGAVSAFVEKTTNSKG
AtCHLH    EDEISALPSILAECVGREIEDVYRGSDKGILSDVELLKEITDASRGAVSAFVEKTTNSKG

          900       910       920       930       940       950
BrCHLH    QVVNVSDKLTSILGFGINEPWVEYLSNTKFYRANRDKLRTVFAFLGECLKLVVMDNELGS
BcCHLH    QVVNVSDKLTSILGFGINEPWVEYLSNTKFYRANRDKLRTVFAFLGECLKLVVMDNELGS
BoCHLH    QVVNVSDKLTSILGFGINEPWVEYLSNTKFYRANRDKLRTVFAFLGECLKLVVMDNELGS
BnaCHLH   QVVNVSDKLTSILGFGINEPWVEYLSNTKFYRANRDKLRTVFAFLGECLKLVVMDNELGS
AtCHLH    QVVDVSDKLTSLLGFGINEPWVEYLSNTKFYRANRDKLRTVFGFLGECLKLVVMDNELGS

          960       970       980       990       1000      1010
BrCHLH    LMQALEGKYVEPGPGGDPIRNPKVLPTGKNIHALDPQAIPTTAAMASAKIVVDRLVERQR
BcCHLH    LMQALEGKYVEPGPGGDPIRNPKVLPTGKNIHALDPQAIPTTAAMASAKIVVDRLVERQR
BoCHLH    LMQALEGKYVEPGPGGDPIRNPKVLPTGKNIHALDPQAIPTTAAMASAKIVVDRLVERQK
BnaCHLH   LMQALEGKYVEPGPGGDPIRNPKVLPTGKNIHALDPQAIPTTAAMASAKIVVDRLVERQK
AtCHLH    LMQALEGKYVEPGPGGDPIRNPKVLPTGKNIHALDPQAIPTTAAMASAKIVVERLVERQK

         1020      1030      1040      1050      1060      1070
BrCHLH    LENEGKYPETIALVLWGTDNIKTYGESLGQVLWMIGARPVADGLGRVNRVEPVSLEELGR
BcCHLH    LENEGKYPETIALVLWGTDNIKTYGESLGQVLWMIGARPVADGLGRVNRVEPVSLEELGR
BoCHLH    MENEGKYPETIALVLWGTDNIKTYGESLGQVLWMIGARPVADGLGRVNRVEPVSLEELGR
BnaCHLH   MENEGKYPETIALVLWGTDNIKTYGESLGQVLWMIGARPVADGLGRVNRVEPVSLEELGR
AtCHLH    LENEGKYPETIALVLWGTDNIKTYGESLGQVLWMIGVRPIADTFGRVNRVEPVSLEELGR
```

```
           1080      1090      1100      1110      1120      1130
BrCHLH     PRIDVVVNCSGVFRDLFINQMNLLDRAIKMVAELDEPVEMNYVRKHAMEQAATLGVDIRE
BcCHLH     PRIDVVVNCSGVFRDLFINQMNLLDRAIKMVAELDEPVEMNYVRKHAMEQAATLGVDIRE
BoCHLH     PRIDVVVNCSGVFRDLFINQMNLLDRAIKMVAELDEPVEMNYVRKHAMEQAATLGVDIRE
BnaCHLH    PRIDVVVNCSGVFRDLFINQMNLLDRAIKMVAELDEPVEMNYVRKHAMEQAATLGVDIRE
AtCHLH     PRIDVVVNCSGVFRDLFINQMNLLDRAIKMVAELDEPVEQNFVRKHALEQAEALGIDIRE

           1140      1150      1160      1170      1180      1190
BrCHLH     AATRVFSNASGSYSSNISLAVENSSWNDEKQLQDMYLSRKSFAFDSDAPGAGMAEKKQVF
BcCHLH     AATRVFSNASGSYSSNISLAVENSSWNDEKQLQDMYLSRKSFAFDSDAPGAGMAEKKQVF
BoCHLH     AATRVFSNASGSYSSNISLAVENSSWNDEKQLQDMYLSRKSFAFDSDAPGAGMAEKKQVF
BnaCHLH    AATRVFSNASGSYSSNISLAVENSSWNDEKQLQDMYLSRKSFAFDSDAPGAGMAEKKQVF
AtCHLH     AATRVFSNASGSYSANISLAVENSSWNDEKQLQDMYLSRKSFAFDSDAPGAGMAEKKQVF

           1200      1210      1220      1230      1240      1250
BrCHLH     EMALMTAEVTFQNLDSSEISLTDVSHYFDSDPTNLVQSLRKDKKKPSAYIADTTTANAQV
BcCHLH     EMALMTAEVTFQNLDSSEISLTDVSHYFDSDPTNLVQSLRKDKKKPSAYIADTTTANAQV
BoCHLH     EMALMTAEVTFQNLDSSEISLTDVSHYFDSDPTNLVQSLRKDKKKPSAYIADTTTANAQV
BnaCHLH    EMALMTAEVTFQNLDSSEISLTDVSHYFDSDPTNLVQSLRKDKKKPSAYIADTTTANAQV
AtCHLH     EMALSTAEVTFQNLDSSEISLTDVSHYFDSDPTNLVQSLRKDKKKPSSYIADTTTANAQV

           1260      1270      1280      1290      1300      1310
BrCHLH     RSLSETVRLDARTKLLNPKWYEGMMSSGYEGVREIEKRLTNTVGWSATSGQVDNWVYEEA
BcCHLH     RSLSETVRLDARTKLLNPKWYEGMMSSGYEGVREIEKRLTNTVGWSATSGQVDNWVYEEA
BoCHLH     RSLSETVRLDARTKLLNPKWYEGMMSSGYEGVREIEKRLTNTVGWSATSGQVDNWVYEEA
BnaCHLH    RSLSETVRLDARTKLLNPKWYEGMMSSGYEGVREIEKRLTNTVGWSATSGQVDNWVYEEA
AtCHLH     RTLSETVRLDARTKLLNPKWYEGMMSSGYEGVREIEKRLSNTVGWSATSGQVDNWVYEEA

           1320      1330      1340      1350      1360      1370
BrCHLH     NTTFIKDEEMLNRLMNTNPNSFRKMIQTFLEANGRGYWETSEDNIEKLKDLYSQVEDKIE
BcCHLH     NTTFIKDEEMLNRLMNTNPNSFRKMIQTFLEANGRGYWETSEDNIEKLKDLYSQVEDKIE
BoCHLH     NTTFIKDEEMLNRLMNTNPNSFRKMIQTFLEANGRGYWETSEDNIEKLKDLYSQVEDKIE
BnaCHLH    NTTFIKDEEMLNRLMNTNPNSFRKMIQTFLEANGRGYWETSEDNIEKLKDLYSQVEDKIE
AtCHLH     NSTFIQDEEMLNRLMNTNPNSFRKMLQTFLEANGRGYWDTSAENIEKLKELYSQVEDKIE

           1380
BrCHLH     GIDRX
BcCHLH     GIDR.
BoCHLH     GIDRX
BnaCHLH    GIDRX
AtCHLH     GIDR.
```
