# Supplementary material for: Convergent roles of BcGUN4.1, BcSG1, BcCHLH, and BcTPR4 in regulating the leaf greenness of non-heading Chinese cabbage
Source: Mol Hortic. 2026 May 8;6:32. doi: 10.1186/s43897-025-00216-5 (PMC13154631; doi:10.1186/s43897-025-00216-5)
Supplement: Supplementary file 3 — Supplementary Material 3: Fig. S3. Regulatory network between SG1 and GUN4 predicted via GeneMANIA. [file 43897_2025_216_MOESM3_ESM.pdf]

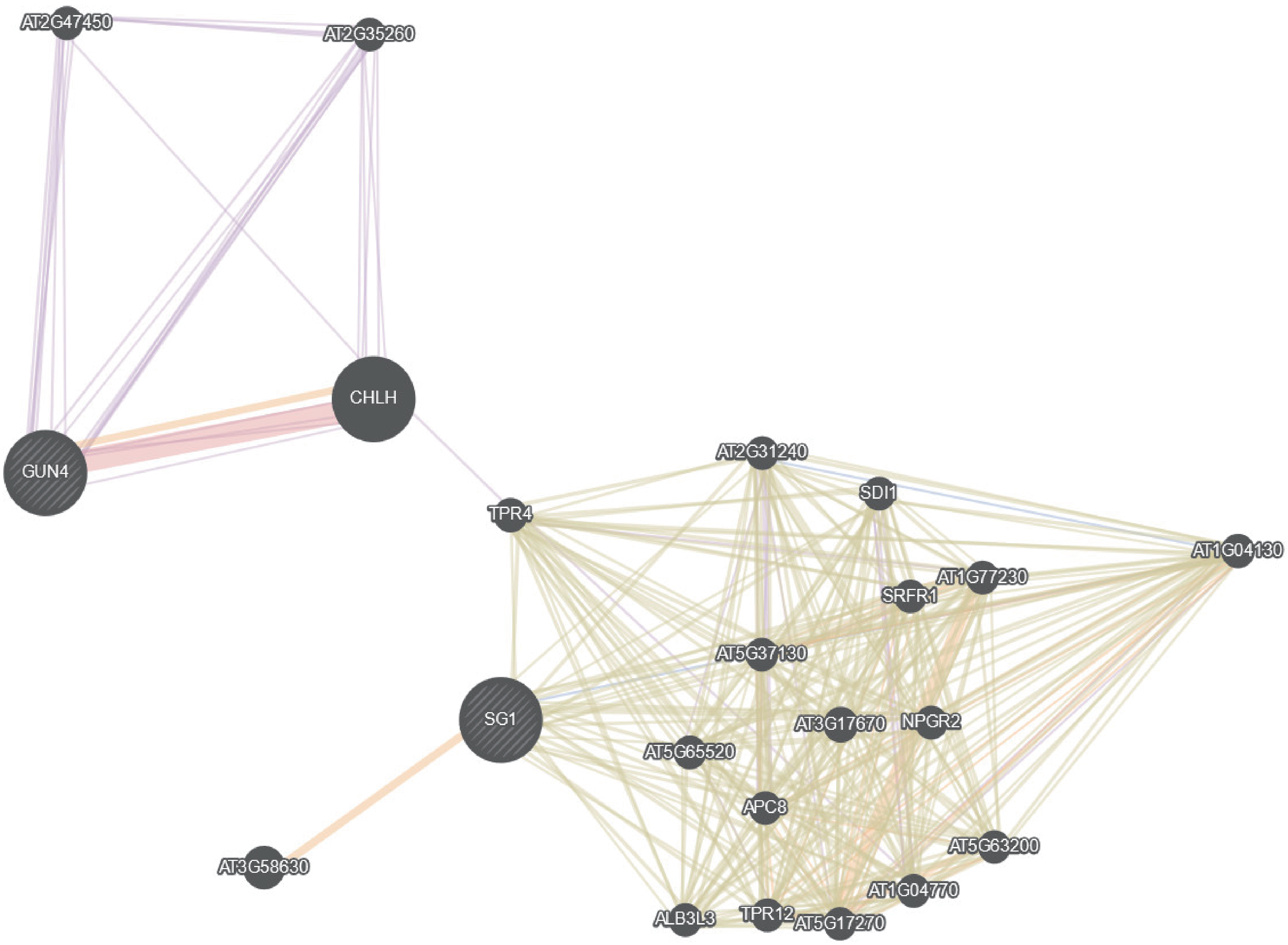

Networks

- Physical Interactions
- Predicted
- Co-expression
- Shared protein domains
- Genetic Interactions
- Co-localization

Fig. S6 The regulatory network between BcSG1 and BcGUN4 predicted by GeneMANIA
